# A chromosome arm from *Thinopyrum intermedium* × *Thinopyrum ponticum* hybrid confers increased tillering and yield potential in wheat

**DOI:** 10.1007/s11032-024-01439-y

**Published:** 2024-01-22

**Authors:** Edina Türkösi, Éva Szakács, László Ivanizs, András Farkas, Eszter Gaál, Mahmoud Said, Éva Darkó, Mónika Cséplő, Péter Mikó, Jaroslav Doležel, Márta Molnár-Láng, István Molnár, Klaudia Kruppa

**Affiliations:** 1 Centre for Agricultural Research, Hungarian Research Network (HUN-REN), 2462 Martonvásár, Hungary; 2 Centre of Plant Structural and Functional Genomics, Institute of Experimental Botany of the Czech Academy of Sciences, 779 00 Olomouc, Czechia; 3 Agricultural Research Centre, Field Crops Research Institute, Cairo, Egypt

**Keywords:** *Agropyron glael*, Yield potential, Tillering, Flow cytometric sorting, GISH, FISH

## Abstract

Tiller number is a key component of wheat plant architecture having a direct impact on grain yield. Because of their viability, biotic resistance, and abiotic stress tolerance, wild relative species are a valuable gene source for increasing wheat genetic diversity, including yield potential. *Agropyron glael*, a perennial hybrid of *Thinopyrum intermedium* and *Th. ponticum*, was created in the 1930s. Recent genome analyses identified five evolutionarily distinct subgenomes (J, J^st^, J^vs^, J^r^, and St), making *A. glael* an important gene source for transferring useful agronomical traits into wheat. During a bread wheat × *A. glael* crossing program, a genetically stable translocation line, WT153397, was developed. Sequential *in situ* hybridizations (McGISH) with J-, St-, and D-genomic DNA probes and pSc119.2, Afa family, pTa71, and (GAA)_7_ DNA repeats, as well as molecular markers specific for the wheat 6D chromosome, revealed the presence of a 6DS.6J^vs^ Robertsonian translocation in the genetic line. Field trials in low-input and high-input breeding nurseries over four growing seasons demonstrated the *Agropyron* chromosome arm’s high compensating ability for the missing 6DL, as spike morphology and fertility of WT153397 did not differ significantly from those of wheat parents, Mv9kr1 and ‘Mv Karizma.’ Moreover, the introgressed 6J^vs^ chromosome arm significantly increased the number of productive tillers, resulting in a significantly higher grain yield potential compared to the parental wheat cultivars. The translocated chromosome could be highly purified by flow cytometric sorting due to the intense fluorescent labeling of (GAA)_7_ clusters on the *Thinopyrum* chromosome arm, providing an opportunity to use chromosome genomics to identify *Agropyron* gene variant(s) responsible for the tillering capacity. The translocation line WT153397 is an important genetic stock for functional genetic studies of tiller formation and useful breeding material for increasing wheat yield potential. The study also discusses the use of the translocation line in wheat breeding.

**Supplementary information:**

The online version contains supplementary material available at 10.1007/s11032-024-01439-y.

## Introduction

Hexaploid wheat (*Triticum aestivum* L., 2*n* = 6*x* = 42, BBAADD) is one of the most important grain crops, grown on about 220 million hectares worldwide (FAOSTAT 2020). To feed the world’s growing population, the current wheat yield production of 763.7 million tons (International Grain Council, 2020/2021) should be increased by 60% until 2050 (https://iwyp.org/global-challenge). However, thousands of years of cultivation and domestication of wheat have led to a reduction in genetic diversity (Tiwari et al. 2014), which has hampered the development of high-yielding cultivars and yield stability under biotic and abiotic stresses (Sears 1981; Jiang et al. 1993; Friebe et al. 1996; Oliver et al. 2006).

Improving wheat structural and reproductive traits contributes to increased crop yield potential (YP) (Reynolds et al. 2011), which is the maximum yield per unit land area achieved under optimal environmental conditions. Among the morphological traits, tiller formation correlates linearly with the number of spikes and has a strong effect on grain yield (Darwinkel 1980). In wheat, two types of tillers form the following: productive tillers, which lead to the formation of spikes and thus significantly contribute to the total number of grains per plant and grain yield, and non-productive tillers, which reduce grain yield (Kirby and Jones 1977; Kulshrestha and Chowdhury 1987).

Species related to wheat are a valuable source of genetic variation for expanding the genetic basis of bread wheat (Tester and Langridge 2010; Mujeeb-Kazi et al. 2013) and improving its morphological and reproductive traits (Reynolds et al. 2011). The introgression of the 1RS chromosome arm from rye (*Secale cereale*, L.) into wheat in the form of 1BL.1RS wheat-rye Robertsonian translocation has been widely used in wheat breeding programs (Rabinovich 1998). This translocation is present in many wheat cultivars worldwide and has improved the yield potential and biotic stress resistance of cultivated wheat (Zeller 1973; Carver and Rayburn 1994; Moreno-Sevilla et al. 1995).

Wheat wild relatives have also long been used in wheat breeding, primarily to improve biotic resistance and abiotic stress tolerance. Pest and disease-resistance genes are involved in over 80% of the beneficial traits introgressed into cultivated species from wild relatives (Hajjar and Hodgkin 2007). Beginning in the late 1970s, CIMMYT produced and characterized interspecific and intergeneric crosses to study genetic relationships and to introgress genetic variation from wild species into wheat (Mujeeb-Kazi and Hettel 1995). Because their homoeologous relationship with wheat is closer than that of other perennial species (King et al. 1993; Mujeeb-Kazi and Hettel 1995; Ceoloni et al. 2014), *Thinopyrum* species were involved in one of the earliest alien gene transfers from wild species into wheat. Wheatgrasses have been used successfully to improve wheat biotic resistance and abiotic stress tolerance. However, only a few studies have been published on the beneficial effect of wild alien chromatin on wheat yield potential (Monneveux et al. 2003; Kuzmanović et al. 2014; Gao et al. 2021). The 7DL.7Ag translocation from *Lophopyrum elongatum* (syn: *Thinopyrum ponticum*), which carries the leaf rust resistance gene *Lr19*, was found to significantly increase grain yield (Monneveux et al. 2003). New knowledge in the genetics of desired traits, increased availability of wild relatives in gene banks, improved interspecific hybridization techniques, and advanced molecular technologies may lead to an expansion of the scale of agronomic traits introgressed from related wild species into cultivated wheat.

The genus *Thinopyrum*, which belongs to the wheat tertiary gene pool, has a variable genome structure ranging from diploid to auto- and allodecaploid species (Ceoloni et al. 2014). For example, the exact genomic constitution of the perennial *Th. intermedium* (Host) Barkworth & D.R. Dewey, a segmental autoallohexaploid (2*n* = 6*x* = 42) species, has shown ambiguous features. Liu and Wang (1993) proposed the genome constitution J^e^J^e^J^e^J^e^SS, with the subgenome J^e^ originating from *Th. elongatum* (Host) D. Dewey (2*n* = 2*x* = 14, EE) and the subgenome S originating from *Pseudoroegneria spicata* (Pursh) A. Löve (2*n* = 2*x* = 14, SS or StSt). Later, it was suggested to rename the S genome with St, since the genomes of the *Aegilops* species in the section Sitopsis were also assigned the S letter. Using genomic in situ hybridization (GISH), Chen et al. (1998) found that signals of the St-genomic probe were located at the centromeric region on 11 out of the 28 J-genome chromosomes, prompting them to rename the genome symbol to JJJ^s^J^s^StSt. According to the findings of Deng et al. (2013), Kruppa and Molnár-Láng (2016), and Cseh et al. (2019), who found only 8 to 10 centromeric St GISH signals, the number of *Th. intermedium* chromosomes with St centromeres varies by genotype. Kishii et al. (2005) suggested that *Dasypyrum villosum* L. (2*n* = 2*x* = 14, VV) was also involved in the evolution of *Th. intermedium* because the V-genomic probe showed strong signals along the length but lacked signals around centromeric regions in several chromosomes and telomeric regions of all chromosomes. Wang et al. (2015) proposed the most recent change to the genomic designation as J^vs^J^r^St, where J^vs^ and J^r^ represent the ancestral genomes of present-day J^b^ of *Th. bessarabicum* (Savul. & Rayss) Á.Löve (2*n* = 2*x* = 14) and J^e^ of *Th. elongatum*, respectively, with J^vs^ being more ancient.

*Th. intermedium* and *Th. ponticum* (Podp.) Z.-W. Liu & R.-C. Wang (2*n* = 10*x* = 70; JJJJJJJ^s^J^s^) are valuable gene sources for wheat since they have useful agronomic and quality traits such as high grain protein content, bread-making quality, resistance to rust diseases, and tolerance to low temperature, drought, moisture, and salinity (Dubcovsky et al. 1994; Tang et al. 2000; Colmer et al. 2006; Garg et al. 2014; Tong et al. 2021).

In order to pyramid genes and QTLs encoding useful agronomic traits of *Th. intermedium* and *Th. ponticum* in a bridge material that can be used as a gene source for introgression breeding of wheat, N. V. Tsitsin developed an artificial hybrid, *Agropyron glael* (2*n* = 8*x* = 56) in the 1930s by crossing *Th. intermedium* (syn. *A. glaucum* (Desf. ex DC.) Roem. & Schult) and *Th. ponticum* (syn. *A. elongatum* (Host) P. Beauv.). A detailed molecular cytogenetic analysis of *A. glael* revealed that it carried 18 J, 28 J^St^, and 8 St chromosomes with J-St and J^St^St translocations and chromosomes with decreased fluorescence intensity, resulting in unique hybridization patterns (Kruppa and Molnár-Láng 2016). The *A. glael* clone No. 8 obtained from the Moscow Main Botanical Garden was used in the wheat introgression breeding program at the Centre for Agricultural Research (Kruppa et al. 2016). In 2001, the winter wheat line ‘Martonvásári 9 kr1’ (Mv9kr1) carrying recessive crossability alleles (*kr1kr1kr2kr2*) (Molnár-Láng et al. 1996) was crossed with *A. glael*. Backcrossing and selfing the hybrid plants resulted in the selection of several partial amphiploid lines (Kruppa et al. 2016), which were then backcrossed with wheat to select addition, substitution, or translocation lines.

The present study reports on the development of a wheat/*A. glael* disomic translocation (WT153397) with positive effects on reproductive tiller formation and grain yield when compared to the wheat parents. Further aims were to (1) identify the chromosome composition of the WT153397 introgression line using molecular cytogenetic, flow cytometric, and molecular marker analyses, and (2) evaluate the effects of the added alien chromosome segment on wheat agronomic performance in field trials conducted over four growing seasons. The study also reports on the inclusion of the translocation line in wheat breeding.

## Materials and methods

### Plant materials

The Mv9kr1 × *A. glael* BC_1_F_1_ partial amphiploid genotype (Kruppa et al. 2016; Kruppa and Molnár-Láng 2016) was backcrossed with the facultative elite wheat cultivar Mv Karizma (Supplementary Figure S1). The wheat/*A.glael* translocation line WT153397 was selected in the BC_3_F_2_ generation using molecular cytogenetic techniques. The WT153397 line, along with the hexaploid wheat crossing partners Mv9kr1, ‘Chinese Spring’ (CS), and ‘Mv Karizma,’ was used for field phenotyping experiments in Martonvásár low-input and high-input nurseries over a 4-year period (2019, 2021, 2022 and 2023). Beginning in 2018, the WT153397 line was involved in a wheat breeding program in Martonvásár. The maternal WT153397 line was crossed with the Martonvásár elite cultivar Mv Nádor, and the offspring were selected based on the plants’ tillering ability and spike length. The phenotype of the F_5_ generation was assessed in the high-input nursery in 2023 and compared to that of the Mv9kr1 wheat line and the cultivar Mv Nádor.

For molecular marker analyses, genomic DNA was isolated from the line WT153397, the wheat genotypes Mv9kr1 and ‘Mv Karizma,’ the parental *A. glael* clone No. 8, the *Aegilops tauschii* (2*n* = 2*x* = 14, DD) accession MvGB605, and the durum wheat (*T. turgidum* ssp*. durum*) cultivar Langdon (2*n* = 4*x* = 28, BBAA).

Genomic DNA isolated from *Th. bessarabicum*, *Pseudoroegneria spicata*, *Aegilops tauschii*, *Secale cereale* L., *Oryza sativa* L., *T. aestivum*, and *T. turgidum* ssp. *durum* was used as probes or blocking DNA for GISH, or as templates to amplify specific DNA sequences for fluorescence *in situ* hybridization (FISH).

### Probe preparation and fluorescence *insitu* hybridization

Mitotic chromosome preparations were made from germinating root tips, as described by Lukaszewski et al. (2004). In order to visualize *Thinopyrum* (*A. glael*) chromatin in the wheat genetic background, the following genomic probes were applied: total genomic DNA from *Th. bessarabicum* was labeled with biotin-11-dUTP (Roche, Mannheim, Germany) and used as J-genomic probe, while DNA from *Ps. spicata* was labeled with digoxigenin-11-dUTP (Roche) or biotin-11-dUTP using random priming labeling and used as St-genomic probe, as described by Kruppa and Molnár-Láng (2016). Digoxigenin-11-dUTP-labeled DNA isolated from *Ae. tauschii* was used as a D-genomic probe.

For the FISH experiments, Afa family and pSc119.2 DNA repeats were amplified from the genomic DNA of *Ae. tauschii* and *S. cereale*, respectively, and labeled using PCR (Nagaki et al. 1995; Contento et al. 2005) with digoxigenin-11-dUTP (Roche) and biotin-16-dUTP (Roche, Mannheim, Germany), respectively. The 18S unit of the 45S rDNA was amplified using PCR from rice genomic DNA (Chang et al. 2010) and labeled with 50% biotin-16-dUTP and 50% digoxigenin-11-dUTP. Microsatellite (GAA)_7_ was amplified from *T. aestivum* genomic DNA and labeled with digoxigenin-11-dUTP (Roche) using PCR (Pedersen and Linde-Laursen 1994). Anti-digoxigenin-rhodamine Fab fragments (Roche) and streptavidine-Alexa Fluor 488 (Molecular Probes, Waltham, USA) were used to detect digoxigenin and biotin, respectively.

Chromosomal composition of the line WT153397 was determined using sequential multicolor genomic *in situ* hybridization (mcGISH) and fluorescence *in situ* hybridizations (FISH). GISH reaction mix (15 µL per slide) containing 50% formamide, 2 × SSC, 10% dextran sulfate, 100 ng each of the J- and St-genomic probes, and 3 µg T*. aestivum* blocking DNA was allowed to hybridize overnight at 42 °C. The hybridization mixes used in the GISH experiments to visualize St- and D-genomic parts of the translocated chromosome contained the same amount of St- and D-genomic probes and durum wheat blocking DNA as described above. GISH signals were documented under a microscope after signal detection and chromosome counterstaining with 2 µg mL^−1^ DAPI (4′,6-diamidino-2-phenylindole, Amersham, Germany). Following washing (3 × 30 min in 4 × SSC Tween, 2 × 5 min 2 × SSC at 25 °C), the slides were re-hybridized with DNA repeat probes.

The protocol for the FISH experiments was modified as follows. The hybridization temperature was 37 °C for probes pSc119.2, Afa family, and pTa71, and 42 °C for probe (GAA)_7_ (in a third step hybridization). The hybridization mixture (30 µL per slide) contained 20 ng of pTa71, 70 ng of each of the pSc119.2 and Afa family probes, and salmon sperm as blocking DNA.

The GISH and FISH hybridization sites were documented using an AxioImager M2 fluorescence microscope (Zeiss, Oberkochen, Germany) equipped with an AxioCam MRm CCD camera (Zeiss, Oberkochen, Germany) and filter sets appropriate for DAPI (Zeiss Filterset 49), Alexa Fluor 488 (Zeiss Filterset 38), and Rhodamine (Zeiss Filterset 20). The images were processed with the AxioVision 4.8.2. software (Zeiss, Oberkochen, Germany).

### Flow cytometric chromosome analysis and sorting

Suspensions of intact mitotic metaphase chromosomes from the line WT153397 were prepared from synchronized root tips cells of young seedlings following Vrána et al. (2016a; 2016b). Prior to flow cytometric analysis, chromosomes in suspension were fluorescently labeled by FISHIS (Giorgi et al. 2013) with oligonucleotide probe 5′-FITC-(GAA)_7_-FITC-3′ (Integrated DNA Technologies, USA) and stained with DAPI. Bivariate flow karyotyping and chromosome sorting were carried out using a FACSAria II SORP flow cytometer and sorter (Becton Dickinson Immunocytometry Systems, San José, USA) as described by Molnár et al. (2016) and Said et al. (2019, 2022). The chromosome samples were analyzed at a rate of 900–1400 particles per second, and bivariate flow karyotypes of FITC vs. DAPI fluorescence were acquired. The sorting window was set to the chromosome population of interest on the dot plots, and chromosomes were flow-sorted at a rate of 15–24 per second. Approximately 3000 chromosomes from each chromosome fraction were flow-sorted onto a microscope slide into a 3 µL drop of PRINS buffer supplemented with 2.5% sucrose (Kubaláková et al. 1997). The chromosome content of the flow-sorted fractions was determined using FISH, as described by Kruppa et al. (2016).

### Molecular marker analysis

A total of 26 simple sequence repeats (SSR) and two conserved ortholog set (COS) markers previously mapped on chromosome 6D were selected from the GrainGenes database (https://wheat.pw.usda.gov/GG3/) and used to characterize the wheat/*A. glael* translocation in the line WT153397. Genomic DNA was extracted from fresh young leaves of wheat genotypes Mv9kr1 and ‘Mv Karizma,’ the parental *A. glael* clone No. 8, the *Ae. tauschii* accession MvGB605, the durum wheat cultivar Langdon, and the WT153397 translocation line using a Quick Gene-Mini80 device (FujiFilm, City, Japan) and a QuickGene DNA tissue kit (FujiFilm) according to the manufacturer’s instructions. The PCR reactions were carried out in an Eppendorf Mastercycler (Eppendorf, City, Germany). Reaction mixes (15 µL) contained 20 ng of template DNA, 1.5 µL of 10 × key reaction buffer (MgCl_2_ final concentration of 1.5 mmol/L), 200 µmol/L of each dNTP, 0.2 µmol/L of forward and reverse primers, and 0.375 U of TEMPase Hot Start DNA Polymerase (VWR International, City, Belgium). The PCR profiles and annealing temperatures of the primer pairs used in the reactions are available in the GrainGenes database (https://wheat.pw.usda.gov/GG3/) and are summarized in Supplementary Table S1. The PCR amplicons were separated using a Fragment Analyzer Automated CE System (Advanced Analytical Technologies, City, USA) equipped with a 96-capillary array cartridge (effective length 33 cm) and analyzed with the PROsize v2.0 software (Advanced Analytical Technologies).

The physical positions of the markers on chromosome 6D were determined *in silico* using a sequence similarity approach. The publicly available primer sequences of the markers (Supplementary Table S1) were used as queries in BLASTn searches (Ensembl Plants release 46; https://plants.ensembl.org/Multi/Tools/Blast) against the reference pseudomolecules of A, B, and D genomes of hexaploid wheat (International Wheat Genome Sequencing Consortium (IWGSC) et al. 2018)*.* The start position of each primer sequence was determined by the best hits obtained on the wheat reference sequence using the BLASTn package of the Blast Command Line Application 2.9.0 (ftp:/ftp.ncbi.nlm.nih.gov/) with the following parameters: -task ‘BLASTn’; -evalue 1e-5; -max target seqs 2; -max hsps 1.

### Field phenotyping experiments and experimental design

Morphological traits of the line WT153397 and the wheat genotypes Mv9kr1, ‘Chinese Spring,’ and ‘Mv Karizma’ were evaluated in a low-input, pesticide-free location (Tükrös nursery, Martonvásár, Hungary; geographic coordinates: 47°18′40″N 18°46′56″E) in the 2018–2019, 2020–2021, 2021–2022 and 2022–2023 growing seasons, as well as in a high-input nursery (Bulgárföld, Martonvásár, Hungary, geographic coordinates: 47°19′39″N, 18°47′01″E) in the 2021–2022 and 2022–2023 seasons. During the 2022–2023 growing season, the phenotype of the WT153397 × ‘Mv Nádor’ F_5_ progeny plants was also assessed. In the low-input Tükrös nursery, 40 grains of each genotype were sown (4 × 1 m rows; 10 grains per row) with a 0.15 m row spacing, whereas in the high-input experiments, 250 grains of each genotype were sown in 2 m^2^ plots (5 × 2 m rows; 50 grains per row) with a row distance of 0.15 m, as previously described by Mikó et al. (2014). The trial plots in the high-input nursery were machine-planted (HEGE-80 plot driller) and hand-harvested. Herbicides, insecticides, and artificial fertilizers were used in the high-input fields when necessary, but fungicides were not. The soil has a clayey chernozem texture and a pH of 7.25. The soil contained 2.8% w/w humus, 210 mg/kg P_2_O_5_, and 210 mg/kg K_2_O. The annual average N intake as NPK combined fertilizer was 120 kg/ha active ingredient (Tóth et al. 2022). Herbicides, fungicides, and insecticides were not used in the low-input nursery. Genotypes were sown close to each other to minimize the confounding effects of soil and microclimatic conditions. Additional growing parameters of the field trial locations are summarized in Supplementary Table S2. The weather conditions varied between the years (Supplementary Table S3) but were typical of the Central-European climate measured over the last 25 years (Vida et al. 2022). The first season had average rainfall but high temperatures, with several heat days. The following two seasons were drier and warmer, especially during the grain-filling period. The last season had the most precipitation and the fewest heat days.

In the high-input nursery, a randomized small-plot complete block design with three replications was used. The plot size was 2 m^2^. The outside rows were always excluded during the analysis. In the low-input nursery, borders were sown at the beginning and end of the previous and next parcels, respectively. For phenotypic characterization, ten typical plants were selected from each genotype in each location and season. Plant height (cm) and tillering (number of tillers per plant) were measured in the field immediately before harvest. The length of the main spike (cm), the number of spikelets per main spike, the number of seeds per main spike, the number of seeds per spikelet, and the number of seeds per plant were determined after harvest. The total number of spikes and the yield of the plots were also measured in the high-input experiment. Seed length and width (mm) as well as thousand grain weight (g) were measured using a MARVIN 5.0 Seed Analyzer (MARViTECH GmbH, Germany). The heading time of each genotype grown in a low-input nursery between 2019 and 2023 was determined as the time between January 1 and the day when 50% of the inflorescences reached the DEV59 developmental stage (Tottman 1987).

### Statistical analysis

Significant differences were calculated from ten biological replicates for each year between the line WT153397 and the wheat genotypes Mv9kr1, ‘Chinese Spring,’ and ‘Mv Karizma,’ as well as between the line WT153397 × ‘Mv Nádor’ F_5_ generation and wheat control genotypes (‘Mv Nádor’ Mv9kr1) using the Tukey’s post hoc test of the IBM SPSS Statistics 20.0 software (SPSS Inc., Chicago, IL, USA).

## Results

### Development and molecular cytogenetic analysis of the line WT153397

**Fig. 1:**
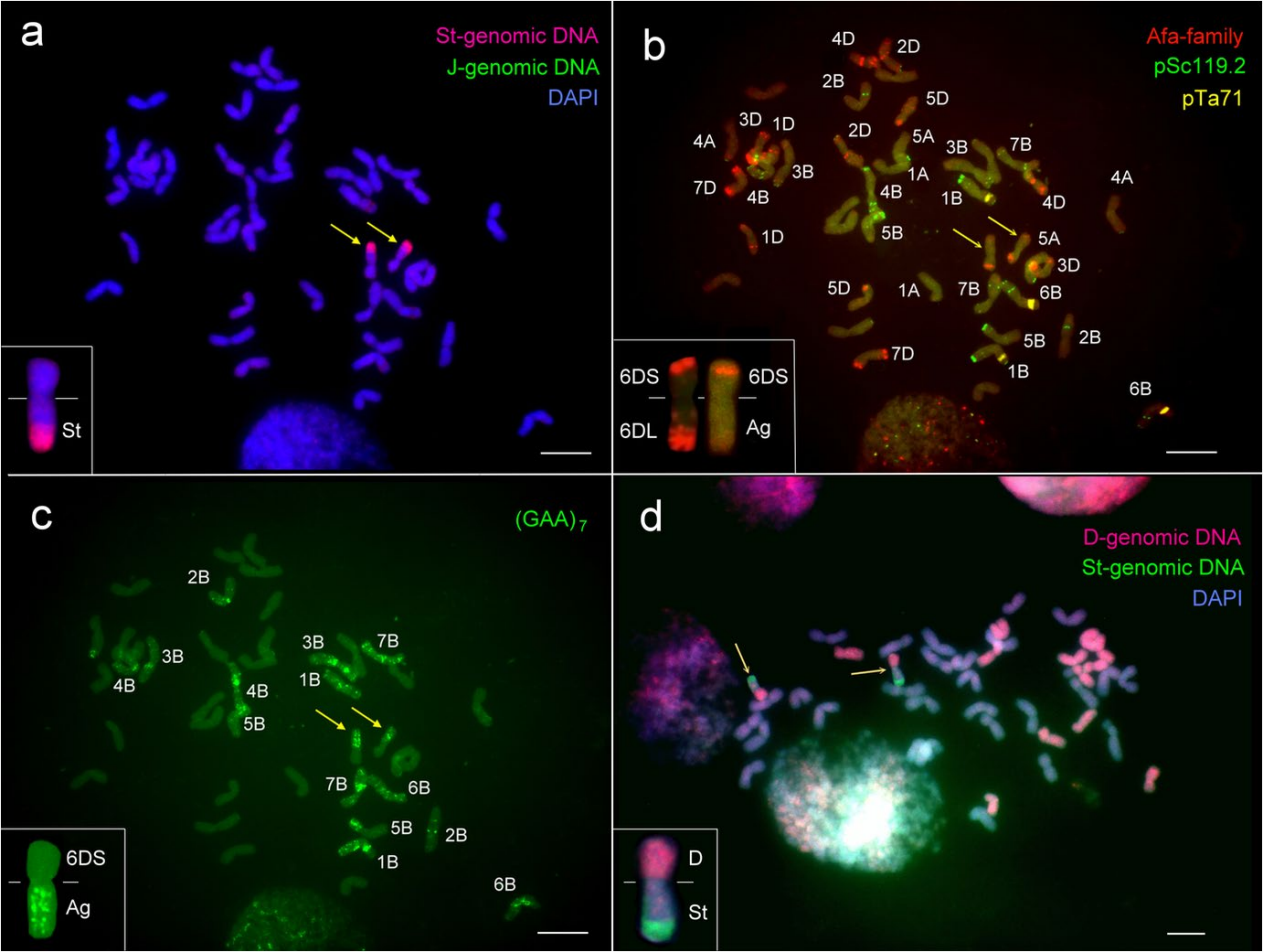
Molecular cytogenetic analysis of the wheat/*A. glael* translocation line WT153397. **a** GISH with *Ps. spicata* St-genomic DNA (red) revealed a disomic wheat/*A. glael* translocation, while biotin-labeled *Th. bessarabicum* J^b^-genomic DNA (green) produced no hybridization signals. The chromosomes were counterstained with DAPI (blue). Inset: the translocated chromosome. **b** FISH on the same mitotic metaphase cell with the Afa family (red), pSc119.2 (green), and pTa71 (yellow) DNA repeat probes. Inset: The FISH pattern of a normal wheat chromosome 6D (left) and the translocated chromosome (right). **c** FISH pattern of the (GAA)_7_ microsatellite probe on the same mitotic cell. Inset: the translocated chromosome with intense GAA clusters on the long arm. **d** McGISH performed with a combination of digoxigenin-labeled D-genomic- (red) and biotin-labeled St-genomic (green) probes, together with A- and B-genomic blocking DNA from *T. turgidum* ssp. *durum.* The red fluorescent signal indicates that only the short arm of the translocation has derived from the wheat D genome. Inset: the translocated chromosome. Scale bars = 10 µm

A schematic representation of the crossing program used to develop wheat/*A. glael* introgression lines is shown in Supplementary Figure S1. Partial amphiploids from the BC_1_F_7_ generation were backcrossed with ‘Mv Karizma’ in two cycles to produce BC_2_F_1_ and BC_3_F_1_ populations. A molecular cytogenetic screening for the presence of *Agropyron* chromosomes and wheat/*Agropyron* translocations was performed on the BC_3_F_2_ generation. Because *A. glael* contains genomes J, J^s^, and St, a combination of digoxigenin-labeled St-genomic probe from *Ps. spicata* (red) and biotin-labeled J-genomic probe from *Th. bessarabicum* (green) was used for *in situ* hybridization. GISH analysis with this probe combination (combination 1) identified a wheat line (WT153397) with 42 chromosomes, including a disomic wheat/*A. glael* translocation chromosome. A red fluorescent signal indicated that a St-genomic segment had been terminally translocated to a wheat chromosome, whereas the absence of green hybridization signals indicated that no J-genomic fragment was involved in the translocation (Fig. 1a).

The slides used for GISH were then subjected to FISH using repetitive DNA probes pSc119.2, Afa family, and pTa71 (Fig. 1b) to identify the wheat chromosome involved in the translocation. The resulting FISH hybridization patterns confirmed the presence of 14 A-genome and 14 B-genome chromosomes. Strong Afa signals also confirmed the presence of one pair for each 1D-5D and 7D chromosome with normal FISH karyotype and the absence of 6D chromosomes. However, a strong Afa signal was detected on the telomeric part of the translocated wheat chromosome arm, which corresponded to the short arm of 6D, suggesting that the *A. glael* chromosome segment was translocated to the 6D wheat chromosome.

In a third step, using the (GAA)_7_ microsatellite probe, intense GAA clusters were detected along the recombinant chromosome arm from the near centromeric region to the telomeric part (Fig. 1c), which is not typical for the D genome (Pedersen and Langridge 1997). This result suggests that the rearranged chromosome arm does not contain D-genome fragments.

To further investigate the structure of the translocation and to visualize its D-genomic part, we used a second combination of biotin-labeled St-genomic probe and digoxigenin-labeled D-genomic probe, as well as A- and B-genomic blocking DNA from *T. turgidum* ssp. *durum*. The GISH analysis revealed that only the short arm of the translocation originated from the D genome, while the long arm originated from *A. glael*, with the terminal part belonging to the St genome (Fig. 1d).

### Flow cytometric analysis and chromosome sorting

The presence of large GAA clusters on the wheat/*A. glael* translocated chromosome was confirmed by flow cytometric analysis and chromosome sorting, in which the chromosome suspension was labeled by FISHIS using a FITC conjugated (GAA)_7_ oligonucleotide probe. The bivariate flow karyotype of wheat/*A. glael* translocation line WT153397 (Supplementary Fig. S2) revealed a chromosome population that is not typical for the standard flow karyotype of hexaploid wheat (Said et al. 2022). The position of the translocated chromosome on the dot plot indicated that its relative DNA content was lower than that of the B-genome chromosomes, but similar to that of the wheat A- and D-genome chromosomes. However, because the *Agropyron* chromosome segment has prominent (GAA)_7_ clusters, its FITC fluorescence was much higher than that of the wheat A- and D-genome chromosomes. These differences allowed us to distinguish the 6D/*A. glael* translocated chromosome from the wheat A-, B-, and D-genome chromosomes (Supplementary Figure S2a). The analysis of 126 chromosomes flow-sorted from the candidate population onto microscopy slides after FISH with pSc119.2, Afa family and pTa71 (Supplementary Figure S2b) confirmed that the translocation could be sorted at high purity (94.4%) with negligible contamination by wheat chromosomes 2D (2.3%), 4A (1.65%) and 4B (1.65%).

### The structure of the wheat/*Agropyron* translocation

The homoeologous relationships between the introgressed *A. glael* chromatin and wheat were also investigated using molecular markers specific for wheat group 6 chromosomes. Twenty-six SSR and two COS markers previously mapped on chromosome 6D were used, and their physical positions on 6D were determined using BLASTn (Supplementary Table S4).

**Fig. 2:**
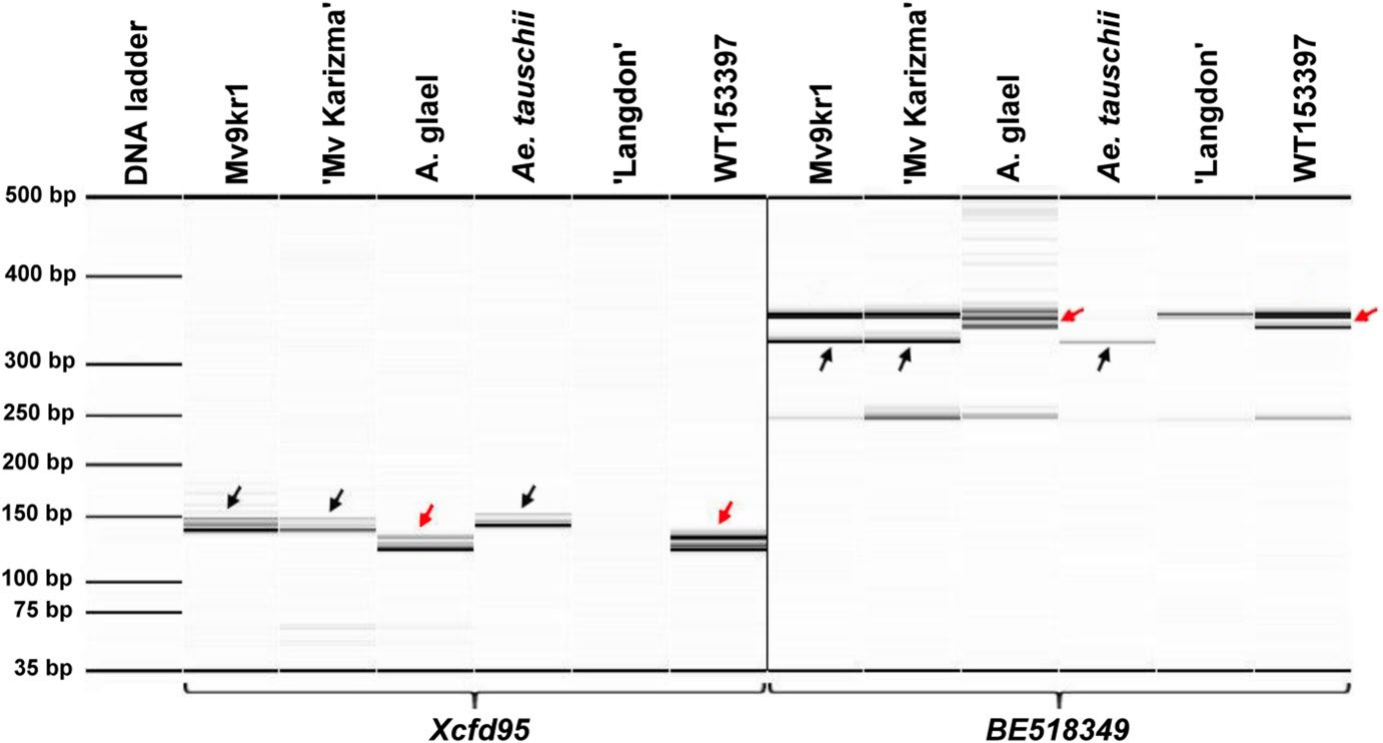
Capillary gel electrophoretogram of 6DL chromosome-specific SSR markers. The markers *Xcfd95* and *BE518349* amplified specific PCR fragments on both the parental wheat genotypes (Mv9kr1 and ‘Mv Karizma’) and *A. glael*. The PCR amplicons specific for 6DL in the parental wheat genotypes and *Ae. tauschii* are indicated by black arrows. The polymorphic fragments (red arrows) amplified on *A. glael* and the line WT153397 indicate that the wheat 6DL is replaced by a chromosome segment from *A. glael* in the translocated chromosome. The control durum wheat cultivar Langdon, which lacks D genome, did not produce 6DL-specific PCR fragments. A 35–500 bp DNA ladder was used as a molecular-weight size marker

**Fig. 3:**
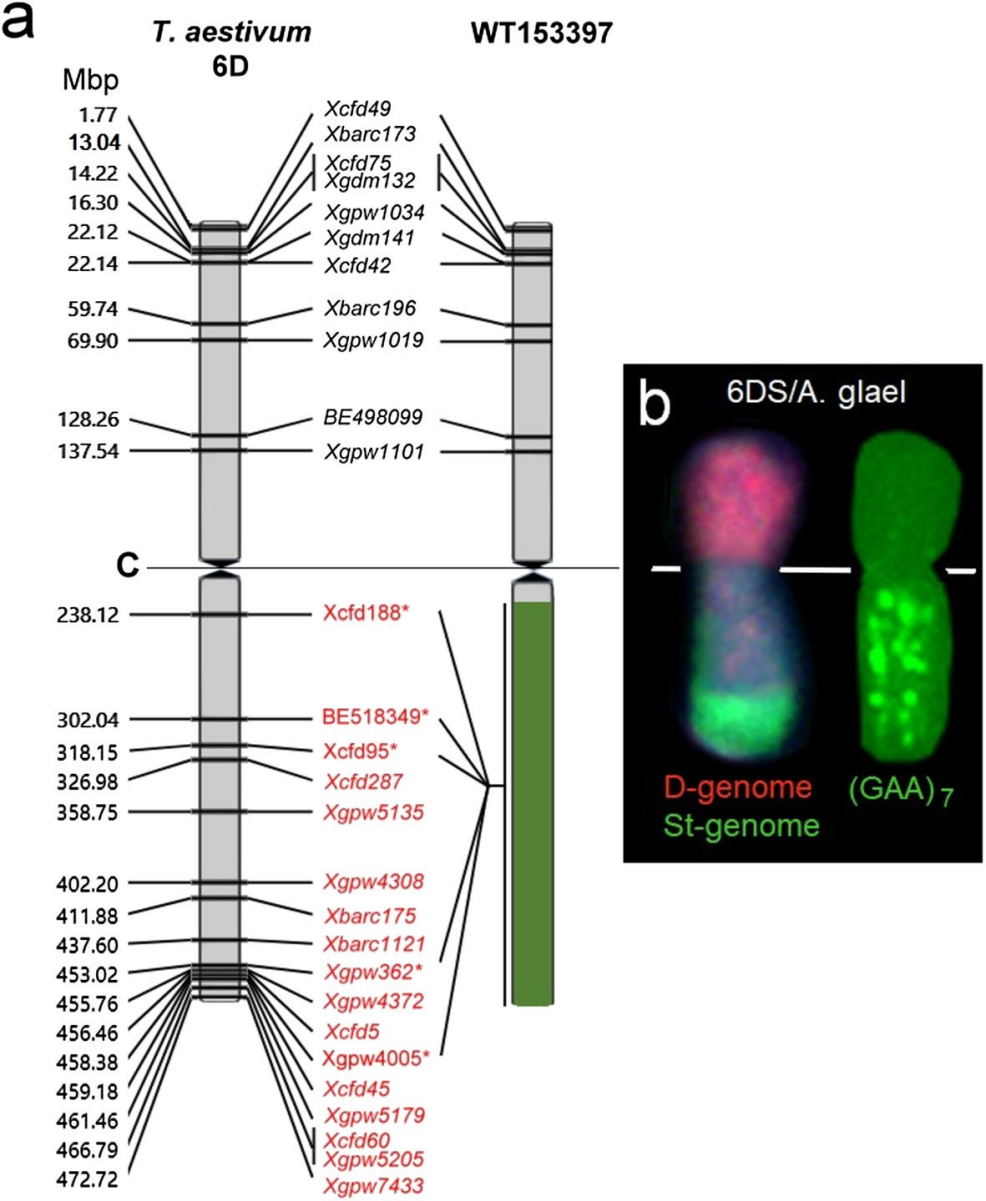
**a** The physical locations of twenty-six SSR and two COS markers on the wheat 6D chromosome and the translocated chromosome of the line WT153397. The 6DS-specific markers are present in both wheat and WT153397, but the 6DL-specific markers are absent from the long arm of the translocated chromosome of WT153397, with the exception of five markers (marked with asterisks) that amplified polymorphic PCR fragments. **b** *In situ* hybridization with the D-genomic probe (red) confirms the presence of the 6DS chromosome arm in the line WT153397, but the intense (GAA)_7_ clusters on the long arm of the translocated chromosome exclude the presence of the 6D long arm. Horizontal lines in Figure a and b mean the centromere (C) position

All 11 markers specific for the short arm of chromosome 6D were detected in the introgression line WT153397, but none of the 17 markers specific for 6DL amplified PCR products on the translocated chromosome, indicating that the long arm of the 6D chromosome was missing from the translocation (Figs. 2 and 3). Five of the 17 markers mapped on the 6DL chromosome arm produced polymorphic fragments between wheat and *A. glael.* All of these markers amplified *Agropyron*-specific products in the line WT153397 suggesting that the missing chromosome arm 6DL was replaced by an *Agropyron* chromosome segment homeologous for wheat group 6 chromosomes (Fig. 3a, b, and c). As a result, the translocated chromosome can be identified as a 6DS.6Ag Robertsonian translocation.

### Morphological traits and yield components of the WT153397 line

**Fig. 4:**
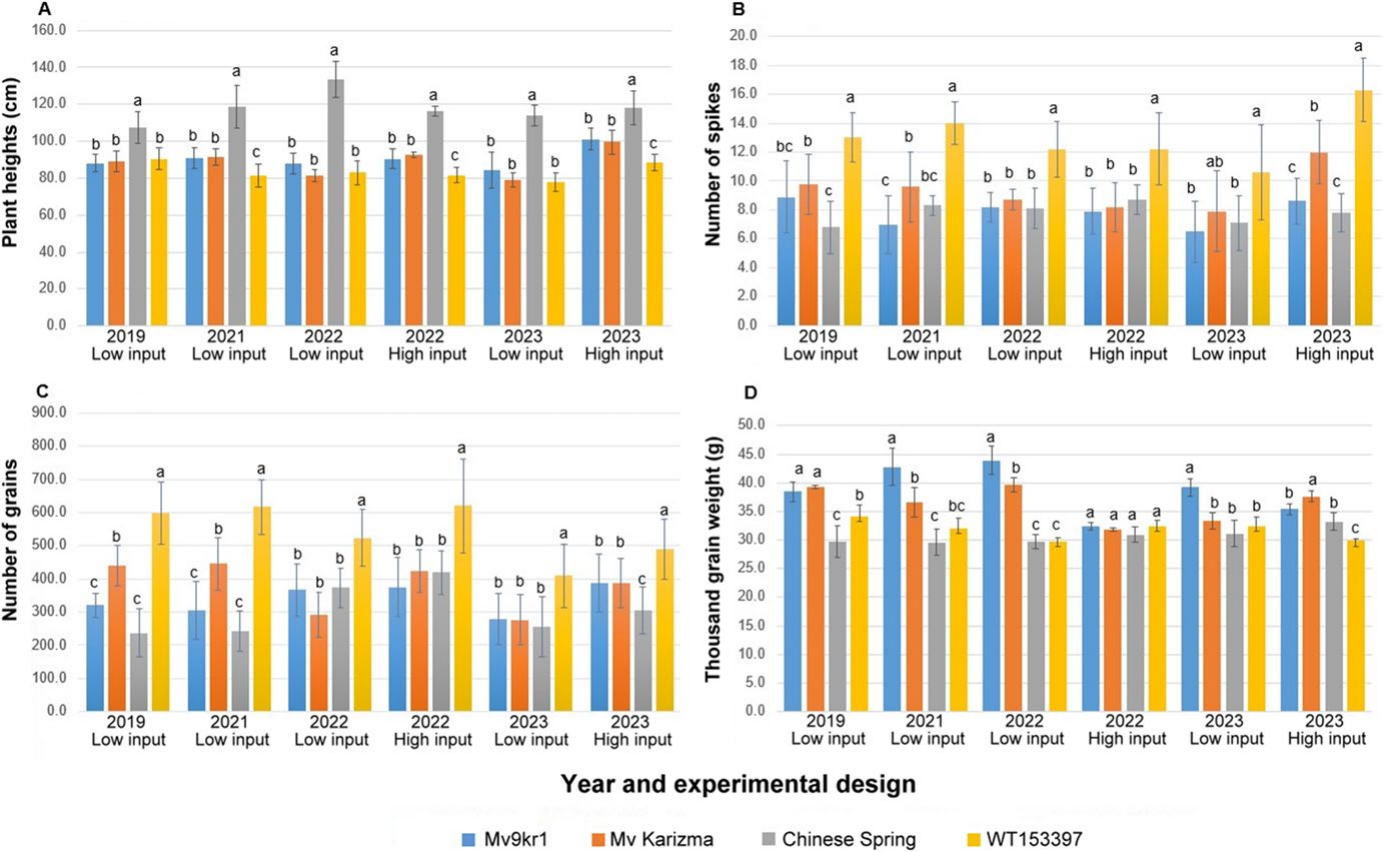
Plant heights (**A**), number of spikes/plant (tillering) (**B**), number of grain/plant (**C**), and thousand-grain weight (**D**) at wheat control genotypes (Mv9kr1, ‘Mv Karizma,’ and ‘Chinese Spring’) and the WT153397 translocation line in four growing seasons (2018–2019, 2020–2021, 2021–2022, and 2022–2023) in low-input and high-input nurseries, Martonvásár, Hungary. Different letters indicate significant differences in Tukey’s post hoc test at *P* < 0.05. Bars: ± standard deviation

**Fig. 5:**
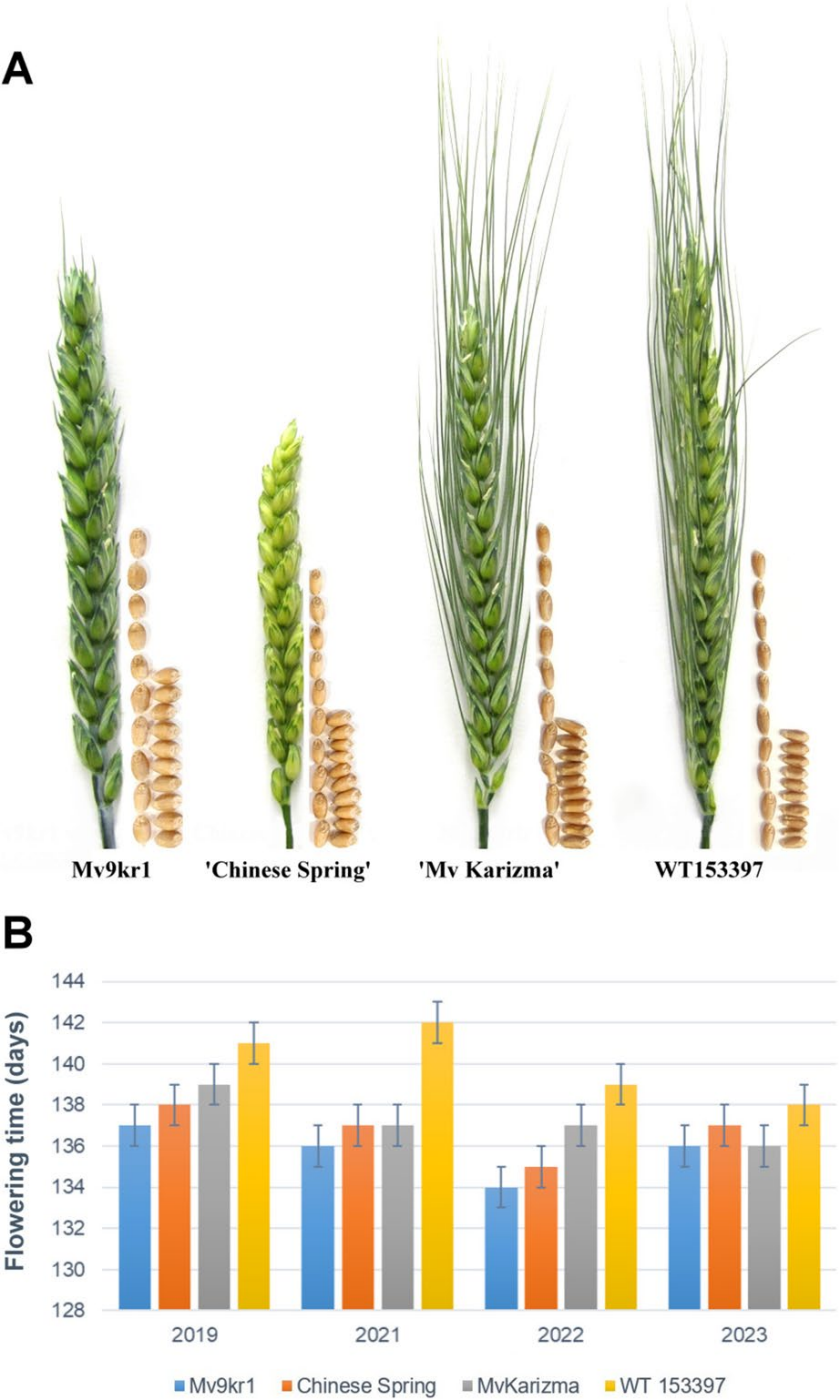
Spike and seed morphology (**A**) and flowering time (**B**) of the parental wheat genotypes Mv9kr1, ‘Chinese Spring’, and ‘Mv Karizma’, and the wheat/A.glael translocation line WT153397 grown in the high-input nursery (spikes were collected in Martonvásár, July 2022) wheat/*A. glael* translocation line WT153397 and the parental wheat genotypes Mv9kr1, ‘Mv Karizma’, and ‘Chinese Spring’ grown in the high-input nursery (spikes were collected in Martonvásár, July 2022)

In order to clarify whether the introgressed *Agropyron* chromosome segment influences wheat morphological traits, the line WT153397 was compared with the parental wheat genotypes Mv9kr1, ‘Chinese Spring,’ and ‘Mv Karizma’ in four field trials (2019, 2021, 2022, and 2023) under low-input, and two trials (2022, 2023) under high-input conditions (Supplementary Table S5 and Fig. 4). The spike and seed morphology of the WT153397 translocation line and the wheat control genotypes are presented in Fig. 5.

The presence of the alien chromosome segment in the new translocation line has no noticeable negative side effects when compared to the morphological and yield components of wheat. The plant height for WT153397 generally did not differ from that of ‘Mv Karizma’ and Mv9kr1, or WT153397 plants were 11–12% shorter in low-input (2021) and high-input (2022) trials (Fig. 4A). On the other hand, Tukey’s post-hoc analysis revealed a high tillering capacity for WT153397, with values ranging from 10.6 to 16.3. This significantly exceeded the number of productive tillers per plant (6.5–12.0) of the wheat genotypes Mv9kr1 and ‘Mv Karizma,’ as well as ‘Chinese Spring’ (6.8–8.7) under both low- and high-input conditions over four growing seasons (Fig. 4B). WT153397 and ‘Mv Karizma’ had a weak tendency for longer spikes and a higher number of seeds per main spike compared to the other wheat genotypes, but differences between WT153397 and the parental wheat genotypes were significant only in the low-input nursery in 2022 (Supplementary Table S5 and Fig. 4). There were no significant differences in the parameters of spikelets per spike and seeds per spikelet between WT153397 and its wheat parents. The increased number of productive tillers and the slightly longer spikes resulted in the highest grain yield (seeds/plant) value of the line WT153397, which was 37–79% higher than the values of ‘Mv Karizma’ under low-input conditions, and 17–46% higher under high-input condition (Supplementary Table S5). This implies that normal sowing density has no effect on the tillering ability and yield potential of the newly developed wheat/*A. glael* translocation line.

There was a tendency for higher TGW of Mv9kr1 relative to ‘Mv Karizma’ because its grains are wider than those of ‘Mv Karizma’ (Figs. 4 and 5, Supplementary Table S6.). The translocation line had the smallest grains with shorter lengths and width. (It is worth noting that the grain width of the translocation did not differ from that of Mv Karizma.) This grain morphological difference of WT153397 was reflected in the smallest TGW. The superior tillering ability of the line WT153397 over Mv9kr1 and ‘Mv Karizma’ resulted in higher yield weight values (g/plant), which was significantly higher in the 2022 high-input and 2023 low-input experiments (Fig. 4D, Supplementary Table S6). The flowering time of WT153397 exceeded that of ‘Mv Karizma’ by 2–5 days in the 4 years studied, indicating that the introgressed *A. glael* chromosome segment has no effect on wheat flowering time.

Because the grain size differed between the two wheat parents Mv9kr1 and Mv Karizma, we wanted to know if the positive effect of the *A. glael* introgression on tillering capacity and yield can be expressed in a different wheat background and whether the small grain size can be increased. To accomplish this, the WT153397 line was crossed with a Hungarian top winter wheat variety ‘Mv Nádor’ as part of a breeding program. The WT153397 × ‘Mv Nádor’ F_5_ progeny was studied on plants grown in the high-input nursery together with the recurrent parental wheat ‘Mv Nádor’ and Mv9kr1 in the 2023 growing season (Supplementary Table S7). The WT153397 × ‘Mv Nádor’ F_5_ plants had a significantly higher tillering ability (almost twice as many spikes/plant), number of seeds/main spike, and fertility (number of seeds/spikelet) than both wheat control genotypes. Moreover, there was no difference in the TGW between the F_5_ plants and ‘Mv Nádor,’ indicating that the smaller TGW observed in WT153397 can be increased in an advanced wheat genetic background.

These morphological traits of the line WT153397 indicate that the 6DS.6Ag translocation is a compensatory type and may be a promising gene source for improving wheat morphology and yield components.

## Discussion

*A. glael* is a promising gene variant reservoir for wheat breeding. The present study is the first to report on a genetically stable introgression line with 42 chromosomes, including a homozygous translocated chromosome carrying a chromosome arm from *A. glael*.

Molecular cytogenetic analyses revealed that wheat chromosome arm 6DS was involved in the translocation. The introgressed *Agropyron* chromosome arm was identified by a terminal St-genomic signal, missing GISH signals with *Th. bessarabicum* DNA and massive GAA clusters from the near centromeric region to the telomere. These results are consistent with those of Cseh et al. (2019), who found that all of the J^vs^-genome chromosomes of *Th. intermedium*, the parental species of *A. glael*, have telomeric hybridization signals with the St-genomic probe, eight of them also have centromeric signals, and the V-genomic probe from *D. villosum* hybridizes with all of them. Giorgi et al. (2013) also reported that the *D. villosum* V-genome chromosomes contain a high number of GAA microsatellite repeats. The strong GAA clusters allowed fluorescent labeling of the V chromosomes in FISHIS using FITC-conjugated GAA oligonucleotide probes and discrimination of the V chromosomes on the bivariate (FITC vs. DAPI) flow karyotype (Giorgi et al. 2013). Previous studies also indicated that the J^vs^ genome preserved repetitive sequences from the *D. villosum* V genome (Kishii et al. 2005; Mahelka et al. 2011; Cseh et al. 2019). It seems reasonable to conclude that GAA microsatellites are common repetitive DNA fractions in *D. villosum* and the *Th. intermedium* J^vs^ genome. A molecular cytogenetic analysis of diploid ancestors of St- and J^b^-genomes, *Ps. spicata*, and *Th. bessarabicum*, respectively, revealed that only one St-genome chromosome contains a GAA signal, while the J^b^ chromosomes do not contain microscopically detectable GAA clusters (Linc et al. 2017).

Based on previous findings and our current results showing the presence of GAA clusters in the introgressed chromosome arm of WT153397, we conclude that the transferred *A. glael* chromosome arm belongs to the J^vs^ genome. Our molecular marker analysis confirmed the presence of the 6DS chromosome arm in the line WT153397, as well as the fact that the introgressed chromosome arm is homoeologous with the missing 6DL. The homoeologous relationship between the diploid *Thinopyrum* genomes and the wheat genome has been established in several publications (Wang et al. 2010; Gaál et al. 2018; Grewal et al. 2018; Baker et al. 2020; Yang et al. 2021). A comparison of *Th. bessarabicum* and wheat chromosomes revealed that the macrosynteny between the two species is preserved, and the majority of *Th. bessarabicum* chromosome arms are collinear with the corresponding A-, B-, and D-genome chromosomes of hexaploid wheat (Grewal et al. 2018). Baker et al. (2020) found that the D genome had the most significant BLAST hits for the markers on the diploid *Th. elongatum* genetic map and that good overall synteny is maintained between wheat and *Th. elongatum*, proving the close relationship between the two genomes. Based on synteny analyses between the *Th. elongatum* and wheat genomes, Yang et al. (2021) also confirmed that the 2A and 2E chromosomes, as well as the 5D and 5E chromosomes, appear to have a good collinearity relationship. Their results agreed with those of Gaál et al. (2018), who used EST sequences to study the macrosyntenic relationship between *Th*. *elongatum* and wheat genomes. Even in polyploid wheatgrasses, the homeology of their genomes with wheat genomes is well preserved.

Kantarski et al. (2017) applied genotyping-by-sequencing (GBS) technology to develop a consensus genetic map for *Th. intermedium*. By aligning the 10,029 mapped markers to the barley reference sequence, significant syntenic relationships between the linkage groups of *Thinopyrum* and barley were found. Later, using an Axiom 35 K Wheat-Relative Genotyping Array to characterize 187 wheat/*Th. intermedium* introgression lines, 634 chromosome-specific SNPs were validated (Cseh et al. 2019). The authors found a significant macrosyntenic relationship between the 21 chromosomes of *Th. intermedium* and their homoeologues from the wheat D genome, including the 6J^vs^-6D synteny. The close 6J^vs^-6D homoeologous relationship is also supported by the present study, as we observed good compensation ability of the introgressed *A. glael* chromosome arm for the missing 6DL for morphological traits and fertility. After four seasons of field trials, there were no significant differences in the spike morphology (spike length, spikelets per spike) and fertility between the introgression line WT153397 and the wheat genotypes Mv9kr1 and ‘Mv Karizma.’

The quality analyses revealed no significant differences in total protein and dietary fiber content (total arabinoxylan), as well as the glutenin/gliadin (Glu/Gli) ratio and unextractable polymeric protein content (UPP%) between the WT153397 line and the wheat parents (data not shown). Because group 6 chromosomes contain genes that determine wheat glutenin and gliadin proteins (Payne et al. 1984, 1987), the similar quality traits of WT153397 support the good compensation ability of 6J^VS^ chromosome arm for the loss of 6DL. However, additional experiments are needed to obtain precise data on the grain chemical composition and bread-making quality of this translocation.

It is important to note that approximately 93.75% of the genes in Mv9kr1 originate from the wheat cultivar Mv9, which adapted well to the agro-ecological conditions of Central Europe (Molnár-Láng et al. 1996), while ‘Mv Karizma’ is a modern elite wheat cultivar registered in 2009. However, field experiments confirmed the translocation line’s outstanding tillering ability, which significantly exceeded the capacity of productive tiller formation in all wheat control genotypes under both low- and high-input conditions.

Tillering is a complex trait determined by the combined effects of multiple genes. Although four single genes (*tin1*, *tin2*, *tin3*, and *ftin*) responsible for tiller inhibition have been identified on wheat chromosomes 1A, 2A, and 3A (Spielmeyer and Richards 2004; Kuraparthy et al. 2007), it was found that a majority of underlying variation for tillering was controlled by quantitative trait loci (QTL) primarily distributed on chromosomes 1A, 2A, 2D, 6A, 6D, 7A, and 7D (Li et al. 2010; Ren et al. 2018; Wang et al. 2018). Our results show that replacing the 6DL chromosome arm with the 6J^vs^ chromosome arm increased the capacity for tiller formation, which is consistent with the findings of Bilgrami et al. (2020). They identified two metaQTLs (MQTL6D-3 and MQTL6D-4) on the terminal region of wheat chromosome 6DL. Our results indicate that QTLs on the 6J^vs^ chromosome arm influence tiller formation more positively than QTLs on the 6DL.

Because the 6DS.6J^vs^ translocated chromosome can be purified at high purity using flow cytometric sorting, identifying key genes on 6J^vs^ responsible for tiller formation using mutant chromosome sequencing (MutChromSeq) will be facilitated. EMS mutagenesis is a suitable method for producing low-tillering wheat mutants, as demonstrated by Dong et al. (2023) for the cloning of a key tiller number regulatory gene, *TN1*. Combining EMS mutagenesis with flow cytometric chromosome sorting and sequencing by MutChromSeq is a powerful approach for rapid gene isolation and identification (Sánchez-Martín et al. 2016), as demonstrated by the cloning of disease resistance genes (Yu et al. 2022; Holden et al. 2022) or genes related to plant morphology (Borrill et al. 2022). The development of low-tillering knock-out EMS mutants, followed by flow sorting and sequencing of the 6DS.6J^vs^ translocated chromosomes from the mutant and wild type WT153397, may be an effective strategy in the future to obtain gene content of 6J^vs^ and identify the candidate gene(s) affecting tillering.

Introgression breeding is hampered by several factors, including the taxonomic relationship between wheat and its relatives, cross-compatibility, fertility of the hybrids, and their offspring (Zhang et al. 2016). The introgression of large alien chromosome segments is also accompanied by the occurrence of the linkage drag phenomenon, which can have a negative impact on grain yield and quality traits (Johansson et al. 2020). However, by using additional backcrosses, appropriate crossing strategies, and selfings, the introgressed chromosome fragment can be shortened, reducing the effect of linkage drag, which causes poorer agronomic features. The translocation line WT153397 was developed through three backcrosses with the recurrent parents, including two backcrosses with ‘Mv Karizma.’ The agronomic characteristics of the translocation line are most similar to ‘Mv Karizma’ in terms of plant height, as well as spike and seed morphology, though the WT153397 line’s spikes are slightly longer and laxer than those of ‘Mv Karizma.’ Its tillering ability and number of seeds per spike were significantly higher than those of the controls in both the low-input and high-input nurseries, indicating that normal sowing density had no effect on the tillering capacity of the line WT153397. The only morphological trait that differed significantly from the wheat controls was the kernel size (thinner grain) and, as a result, the smaller TGW. The fact that the grain width and TGW increased in the ‘Mv Nádor’ genetic background, as observed in the WT153397 × ‘Mv Nádor’ F_5_ lines, supports the possibility that the smaller grain width of the line WT153397 originated from ‘Mv Karizma.’ However, larger-scale field trials should be carried out in the future to support the positive contribution of higher tillering capacity of the 6DS.6J^vs^ translocation to higher wheat yield formation. Mapping and cloning of the *Agropyron* gene responsible for improved tillering will also aid wheat breeding programs in utilizing this gene source.

## Conclusion

Wheat/*Thinopyrum* introgressions can be used to improve not only stress resistance but also yield components of bread wheat. The translocation line WT153397 is genetically stable, with no evidence of linkage drag. The presence of the *A.glael* 6J^vs^ chromosome arm in subsequent generations can be tracked using specific molecular markers as well as molecular cytogenetic methods. According to preliminary results from environments with low and high nutrient supplies, this translocation can even develop reproductive tillers with a 35–45% higher efficiency. The WT153397 × ‘Mv Nádor’ F_5_ plants demonstrated that the improved tillering caused by the 6D.S6J^vs^ translocation chromosome can be combined with larger seed size in the advanced wheat genetic background, potentially resulting in higher yield potential. In the future, large-scale field trials and precise breeding programs will be needed to confirm this finding and investigate end-use quality and other agronomic traits. These advantages enable this line to be used in wheat breeding programs as well as functional genomic studies aiming to clarify the genetic control of reproductive tiller formation.

### Supplementary information

Below is the link to the electronic supplementary material.

Supplementary file1 (DOCX 2761 KB)

## Data Availability

All data generated or analyzed during this study are included in this article and its supplementary information file. Seeds of the described translocation line are freely available to any researcher wishing to use them for non-commercial purposes.
