# Supplementary material for: A chromosome arm from Thinopyrum intermedium × Thinopyrum ponticum hybrid confers increased tillering and yield potential in wheat
Source: Mol Breed. 2024 Jan 22;44(2):7. doi: 10.1007/s11032-024-01439-y (PMC10803699; doi:10.1007/s11032-024-01439-y)
Supplement: Supplementary file 1 — Supplementary file1 (DOCX 2761 KB) [file 11032_2024_1439_MOESM1_ESM.docx]

**SUPPLEMENTARY MATERIALS**

Article title:

**A chromosome arm from *Thinopyrum intermedium* × *Thinopyrum ponticum* hybrid confers increased tillering and yield potential in wheat**

Journal name: **Molecular Breeding**

Authors:

Edina Türkösi^1,†^, Éva Szakács^1,†^, László Ivanizs^1^, András Farkas^1^, Eszter Gaál^1^, Mahmoud Said^2,3^, Éva Darkó^1^, Mónika Cséplő^1^, Péter Mikó^1^, Jaroslav Doležel^2^, Márta Molnár-Láng^1^, István Molnár^1^*, Klaudia Kruppa^1^

Affiliations

^1^ Agricultural Institute, Centre for Agricultural Research, Hungarian Research Network (HUN-REN) 2462 Martonvásár, Hungary;

^2^ Institute of Experimental Botany of the Czech Academy of Sciences, Centre of Plant Structural and Functional Genomics, 779 00 Olomouc, Czechia

^3^ Field Crops Research Institute, Agricultural Research Centre, Cairo, Egypt

^†^ These authors contributed equally to this work.

*Corresponding author: molnar.istvan@hun-ren.atk.hu, Orcid ID: 0000-0002-7167-9319, phone: [+3622569504](tel:%2B3622569504)

**Supplementary Fig. S1** Development of the wheat/*A. glael* translocation line WT153397. The *in situ* hybridization experiments were carried out in the BC_3_F_2_ and subsequent generations. The plants were phenotypically characterized in the BC_3_F_4_-F_6_ generations. ⊗: self-fertilization.

**Supplementary Fig. S2** Flow cytometric analysis and sorting of the wheat/*A. glael* chromosome translocation from the WT153397 genotype. (a) Bivariate flow karyotype FITC vs. DAPI fluorescence of the line WT153397. The translocated chromosomes were flow sorted in high purity (94.4%) using the sort window shown as red rectangle. Inset: flow sorted wheat/A.glael translocation. (b) Example of translocated chromosomes sorted onto a microscope slide after FISH with probes pSc119.2 (green – not visible-), Afa family (red) and 45S rDNA (yellow– not visible-).

**Supplementary Table S1** Details of markers used for PCR experiments

**Supplementary** **Table S2** Growing conditions of plants investigated in the experiments of the present work

^1^= precipitation in the period from sowing to harvest; ^2^from flowering until final dry weight; ^3^daily maximum temperature ≥ 30°C

**Supplementary Table S3** Wheather conditions of the terroirs where plants investigated were grown (during 4 growing seasons, between 2019-2023) ^1^= in the period from sowing to harvest; ^2^from flowering until final dry weight; ^3^daily maximum temperature ≥ 30°C

**Supplementary Table S4** PCR amplification by 6D-specific SSR and COS markers on the bread wheat genotypes Mv9kr1 and 'Mv Karizma,' as well as *A. glael* and the translocation line WT153397

**Supplementary Table S5** Morphological traits of the wheat/*A.glael* WT153397 line and the parental wheat genotypes Mv9kr1, ‘Mv Karizma’, and ‘Chinese Spring’ in four growing seasons (years of harvesting 2019, 2021, 2022 and 2023) and in two locations (low- and high input). Values are the means ± standard deviations of 10 measurements. Values with the same letters in a column do not differ significantly by Tukey’s post hoc test (α = 0.05). Values of WT153397 significantly different from the wheat genotypes have been highlighted by bold letters.

**Supplementary Table S6** Average grain width (in mm), average grain length (in mm), and thousand kernel weight (in grams) of two wheat parental genotypes (Mv9kr1 and Mv Karizma) and the WT153397 translocation line in the four growing seasons. Values are the means ± standard deviations of 3 measurements. Values with the same letters in a column per year do not differ significantly by Tukey’s post hoc test (α = 0.05).

**Supplementary Table S7** Morphological traits of the Mv9kr1wheat line, Mv Nádor elite wheat cultivar and WT153397 × Mv Nádor offsprings (F_5_ generation) in 2022-2023 growing season in high-input nursery. Values are the means ± standard deviations of 10 measurements. Values with the same letters in a column do not differ significantly by Tukey’s post hoc test (α = 0.05). Values of WT153397 significantly different from the wheat genotypes have been highlighted by bold letters.

**Supplementary Fig. S1** Development of the wheat/*A. glael* translocation line WT153397. The *in situ* hybridization experiments were carried out in the BC_3_F_2_ and subsequent generations. The plants were phenotypically characterized in the BC_3_F_4_-F_6_ generations. ⊗: self-fertilization.


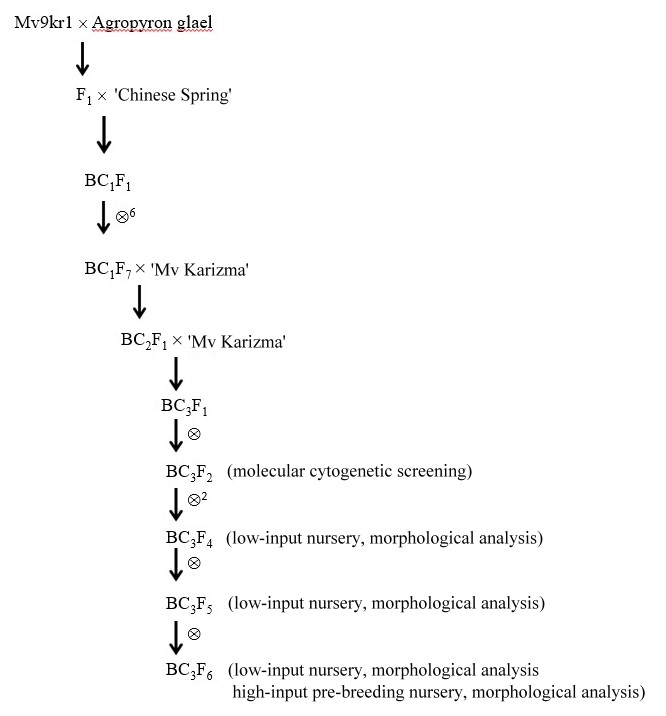


**Supplementary Fig. S2** Flow cytometric analysis and sorting of the wheat/*A. glael* chromosome translocation from the WT153397 genotype. (a) Bivariate flow karyotype FITC vs. DAPI fluorescence of the line WT153397. The translocated chromosomes were flow sorted in high purity (94.4%) using the sort window shown as red rectangle. Inset: flow sorted wheat/A.glael translocation. (b) Example of translocated chromosomes sorted onto a microscope slide after FISH with probes pSc119.2 (green – not visible-), Afa family (red) and 45S rDNA (yellow– not visible-).


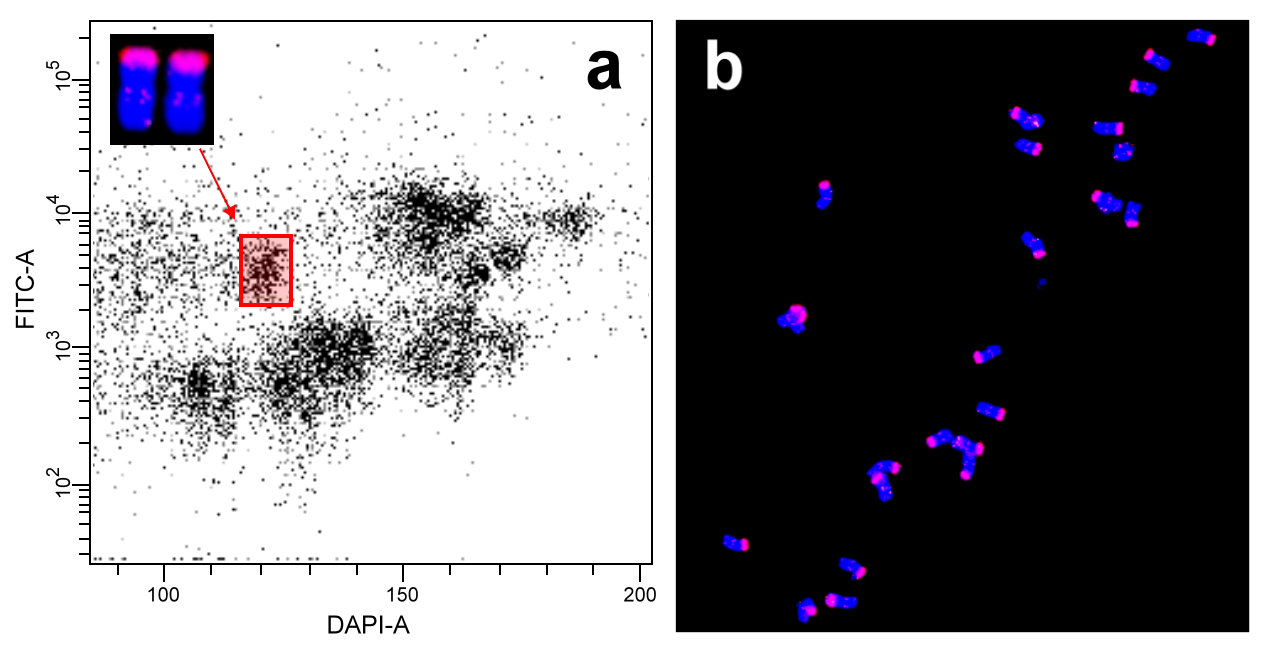


**Supplementary Table S1** Details of markers used for PCR experiments

| Marker name | Forward primer (5’-3’) | Reverse primer (5’-3’) | Ta (°C) |
| --- | --- | --- | --- |
| *Xcfd49* | TGAGTTCTTCTGGTGAGGCA | GAATCGGTTCACAAGGGAAA | 60 |
| *Xbarc173* | GGGGATCCTTCAACAATAACA | GCGAGATGGCATTTTTAAATAAAGAGAC | 50 |
| *Xcfd75* | GCATAAACTTGGACCCTGGA | GCTAAGCCACGCTACCACTC | 60 |
| *Xgdm132* | ACCGCTCGGAGAAAATCC | AGGGGGGCAGAGGTAGG | 60 |
| *Xgpw1034* | TCGCCTGCCTAGTAAGCATT | TATCCCGTTGCAAGCTATCC | 60 |
| *Xgdm141* | ATGGAGACCATGGACCAGAG | GGCGGTGTTCCTATGCC | 60 |
| *Xcfd42* | AGGTTCTAGGGGGCATGTCT | GCTCTCAATGACTGCACTGG | 60 |
| *Xbarc196* | GGTGGGTTTTATCGAATAGATTTGCT | GCGTTTCGTCAAGATTAATGCAGGTTT | 50 |
| *Xgpw1019* | AATTCGATGACTTTGGGGTG | AAGCCCAGGCTAATTCTGGT | 60 |
| *BE498099* | ATCTGTTTACTGCGCGTTCC | CACGGACAAAACAGAAGGAC | 60 |
| *Xgpw1101* | ATCCACTGTCCATCGGTTTC | CAGCGTCTGTCTCCCAATTC | 60 |
| *Xcfd188* | AATGGCTTCACTGTTTGCCT | AAATGGTCCCAGCATTCAAG | 60 |
| *BE518349* | ATCGGGTACAAGCACCAGTC | GCCGCAGATGGCGCCGAGGC | 60 |
| *Xcfd95* | AATCCTGACTTTAAAGCCTTTCC | CATCTGTATGATATTTTGGAGGTCA | 60 |
| *Xcfd287* | TCAAGAAGATGCGTTCATGC | GGGAGCTTTCCCTAGTGCTT | 60 |
| *Xgpw5135* | TTTGTAAGTTGCCTGTGACCC | GGTGGAGACAACGACTGGC | 60 |
| *Xgpw4308* | GATCTGTTTCTTGTTCGCTGG | TGGCCGCGTATGTTAGAAG | 60 |
| *Xbarc175* | GCGTAACAGAAGCGGAGAAAGC | GCGAATCATTTAGTGTTAGGTGGCAGTG | 55 |
| *Xbarc1121* | GCGAGCAAACTGATCCCAAAAAG | TATCGGTGAGTACGCCAAAAACA | 52 |
| *Xgpw362* | GAATCGTCAATGCCCCTCTA | CCCTCTATCGGTCTTTGCTG | 60 |
| *Xgpw4372* | GAGGGAGTAGACATGGTCGC | GAACAAAGCGGGAGTGACAT | 60 |
| *Xcfd5* | TGCCCTGTCCACAGTGAAG | TTGCCAGTTCCAAGGAGAAT | 60 |
| *Xgpw4005* | GGTTCACCTCAATAATCGGC | GTTCTGGTACGGGTCAATGG | 60 |
| *Xcfd45* | TCTCTCCAGTTGCTCCTCGT | ATGTGGAACCGGTCTACTCG | 60 |
| *Xgpw5179* | CCATTCCGCAAATGATGATA | GCGTATTCGGGTTGTTCATT | 60 |
| *Xcfd60* | TGACCGGCATTCAGTATCAA | TGGTCACTTTGATGAGCAGG | 60 |
| *Xgpw5205* | ACGGGTCATATTCCTTGGG | ATGGTCACTTTGATGAGCAGG | 60 |
| *Xgpw7433* | GTACATGGAAGAGACCACACCA | CGCTGAGCAAGGACGATAG | 60 |

Ta: annealing temperature

**Supplementary Table S2** Growing conditions of plants investigated in the experiments of the present work

| Growing conditions | | TRIAL Location | |
| --- | --- | --- | --- |
|  |  | LOW input pre-breeding nursery | HIGH input breeding nursery |
| Location | Geographic coordinates in Martonvásár (Hungary) | 47.311461 N, 18.782787 E | 47.330237 N, 18.784375 E |
|  | Altitude | 109 m | 115 m |
|  | Soil type | chernozem | chernozem |
|  | Fungicide | - | - |
|  | Herbicides, insecticides and artificial fertilizers | - | + |

^1^= precipitation in the period from sowing to harvest; ^2^from flowering until final dry weight; ^3^Daily maximum temperature ≥ 30°C

**Supplementary Table S3** Wheather conditions of the terroirs where plants investigated were grown (during 4 growing seasons, between 2019-2023)

| Weather conditions | | | | | |
| --- | --- | --- | --- | --- | --- |
|  | Mean temperature (˚C) | | | Precipitation (mm) | |
| Growing season | Total^1^ | Grain filling period^2^ | Number of heat days^3^ | Total^1^ | Grain filling period^2^ |
| 2018-2019 | 8.88 | 19.9 | 11 | 356 | 117 |
| 2020-2021 | 8.02 | 24.4 | 14 | 291 | 70 |
| 2021-2022 | 8.29 | 20.8 | 9 | 293 | 78 |
| 2022-2023 | 8.96 | 19.3 | 5 | 430 | 105 |

^1^= in the period from sowing to harvest; ^2^from flowering until final dry weight; ^3^Daily maximum temperature ≥ 30°C

**Supplementary Table S4** PCR amplification by 6D-specific SSR and COS markers on the bread wheat genotypes Mv9kr1 and 'Mv Karizma,' as well as A. glael and the translocation line WT153397

| Marker name | Location | Position on 6D (Mbp) | Amplicons | | | |  |
| --- | --- | --- | --- | --- | --- | --- | --- |
|  |  |  | Bread wheat | | A. glael | WT153397 (wheat/A. glael) | |
| *Xcfd49* | 6DS | 1.77 | + | + | | +/- |  |
| *Xbarc173* | 6DS | 13.04 | + | + | | +/- |  |
| *Xcfd75* | 6DS | 14.22 | + | - | | +/- |  |
| *Xgdm132* | 6DS | 14.22 | + | + | | +/- |  |
| *Xgpw1034* | 6DS | 16.30 | + | + | | +/- |  |
| *Xgdm141* | 6DS | 22.12 | + | + | | +/- |  |
| *Xcfd42* | 6DS | 22.14 | *+* | *+* | | *+/-* |  |
| *Xbarc196* | 6DS | 59.74 | *+* | *-* | | *+/-* |  |
| *Xgpw1019* | 6DS | 69.90 | *+* | *+* | | *+/-* |  |
| *BE498099* | 6DS | 128.26 | *+* | *+* | | *+/-* |  |
| *Xgpw1101* | 6DS | 137.54 | *+* | *+* | | *+/-* |  |
| *Xcfd188** | 6DL | 238.12 | + | + | | **-/+** |  |
| *BE518349** | 6DL | 302.04 | + | + | | **-/+** |  |
| *Xcfd95** | 6DL | 318.15 | + | + | | **-/+** |  |
| *Xcfd287* | 6DL | 326.98 | + | - | | -/- |  |
| *Xgpw5135* | 6DL | 358.75 | + | - | | -/- |  |
| *Xgpw4308* | 6DL | 402.20 | + | - | | -/- |  |
| *Xbarc175* | 6DL | 411.88 | + | - | | -/- |  |
| *Xbarc1121* | 6DL | 437.60 | + | - | | -/- |  |
| *Xgpw362** | 6DL | 453.02 | + | + | | -/+ |  |
| *Xgpw4372* | 6DL | 455.76 | + | - | | -/- |  |
| *Xcfd5* | 6DL | 456.46 | + | - | | -/- |  |
| *Xgpw4005** | 6DL | 458.38 | + | + | | **-/+** |  |
| *Xcfd45* | 6DL | 459.18 | + | - | | -/- |  |
| *Xgpw5179* | 6DL | 461.46 | + | - | | -/- |  |
| *Xcfd60* | 6DL | 466.79 | + | - | | -/- |  |
| *Xgpw5205* | 6DL | 466.79 | + | - | | -/- |  |
| *Xgpw7433* | 6DL | 472.72 | + | + | | -/- |  |

+: the marker amplified a specific PCR fragment (or fragments)

-: the marker did not amplify a specific PCR fragment (or fragments)

*: represents markers that amplified A. glael-specific (polymorph) PCR products in the WT153397 line

**Supplementary Table S5** Morphological traits of the wheat/*A.glael* WT153397 line and the parental wheat genotypes Mv9kr1, ‘Mv Karizma’, and ‘Chinese Spring’ in four growing seasons (2019, 2021, 2022 and 2023) and in two locations (low- and high-input). Values are the means ± standard deviations of 10 measurements. Values with the same letters in a column do not differ significantly by Tukey’s post hoc test (α = 0.05). Values of WT153397 significantly different from the wheat genotypes have been highlighted by bold letters.

| **Field trial** | **Genotype** | **Plant height (cm)** | **Number of spikes/plant** | **Length of main spike (cm)** | **Number of Spikelets/main spike** | **Number of Seeds/main spike** | **Number of Seeds/plant** | **Fertility (seeds/spikelet)** |
| --- | --- | --- | --- | --- | --- | --- | --- | --- |
| **Low-input. 2019** | **Mv9kr1** | 88.1 ± 4.72 b | 8.9 ± 2.46 bc | 10.5 ± 1.08 b | 21.1 ± 1.13 a | 43.5 ± 5.42 ab | 321.0 ± 36.09 c | 2.09 ± 0.30 ab |
|  | **Mv Karizma** | 89.0 ± 5.56 b | 9.8 ± 2.08 b | 11.2 ± 0.79 ab | 21.0 ± 1.41 ab | 47.4 ± 7.52 a | 439.1 ± 60.7 b | 2.28 ± 0.20 a |
|  | **CS** | 107.4 ± 8.63 a | 6.8 ± 1.83 c | 8.6 ± 0.64 c | 19.2 ± 1.46 b | 36.0 ± 10.41 b | 237.7 ± 72.5 c | 1.84 ± 0.42 b |
|  | **WT153397** | 90.6 ± 6.15 b | **13.0 ± 1.73 a** | 11.5 ± 0.70 a | 22.0 ± 1.84 a | 44.6 ± 5.24 ab | **599.3 ± 94.0 a** | 2.47 ± 0.25 a |
| **Low-input. 2021** | **Mv9kr1** | 91.0 ± 5.46 b | 7.0 ± 2.00 c | 10.6 ± 0.74 b | 21.1 ± 1.13 b | 43.5 ± 5.42 a | 303.7 ± 87.35 c | 2.06 ± 0.22 a |
|  | **Mv Karizma** | 91.6 ± 4.40 b | 9.6 ± 2.37 b | 11.8 ± 1.18 a | 21.0 ± 1.41 b | 47.4 ± 7.37 a | 445.3 ± 80.27 b | 2.25 ± 0.31 a |
|  | **CS** | 118.7 ± 11.61 a | 8.3 ± 0.67 bc | 8.6 ± 0.80 c | 23.4 ± 1.42 a | 32.1 ± 10.97 b | 243.1 ± 59.82 c | 1.37 ± 0.44 b |
|  | **WT153397** | **81.4 ± 2.72 c** | **14.0 ± 1.49 a** | 11.2 ± 1.09 ab | 22.2 ± 1.72 ab | 44.8 ± 4.97 a | **616.4 ± 81.94 a** | 2.01 ± 0.15 a |
| **Low-input. 2022** | **Mv9kr1** | 87.8 ± 5.47 b | 8.2 ± 1.07 b | 9.0 ± 1.07 c | 17.9 ± 1.64 b | 47.3 ± 6.82 bc | 366.7 ± 78.0 b | 2.64 ± 0.26 ab |
|  | **Mv Karizma** | 81.3 ± 3.37 b | 8.7 ± 0.74 b | 10.1 ± 0.45 b | 19.2 ± 0.74 b | 39.0 ± 2.75 c | 291.5 ± 68.2 b | 2.03 ± 0.20 c |
|  | **CS** | 133.5 ± 9.82 a | 8.1 ± 1.42 b | 8.4 ± 0.53 c | 23.2 ± 0.87 a | 52.6 ± 3.74 b | 373.0 ± 59.6 b | 2.32 ± 0.27 bc |
|  | **WT153397** | 83.0 ± 6.40 b | **12.2 ± 1.86 a** | **12.2 ± 0.95 a** | 22.8 ± 1.66 a | **61.9 ± 11.5 a** | **524.0 ± 86.3 a** | 2.70 ± 0.39 a |
| **High-input. 2022** | **Mv9kr1** | 90.6 ± 5.23 b | 7.9 ± 1.60 b | 11.1 ± 0.84 b | 24.1 ± 1.75 ab | 58.1 ± 9.40 b | 375.3 ± 89.7 b | 2.39 ± 0.35 b |
|  | **Mv Karizma** | 92.7 ± 1.73 b | 8.2 ± 1.66 b | 13.3 ± 0.97 a | 23.9 ± 1.22 ab | 67.5 ± 10.80 ab | 422.9 ± 64.2 b | 2.61 ± 0.90 ab |
|  | **CS** | 116.3 ± 2.83 a | 8.7 ± 0.97 b | 8.9 ± 0.84 c | 22.0 ± 2.44 b | 69.7 ± 9.12 ab | 419.0 ± 66.3 b | 3.19 ± 0.47 a |
|  | **WT153397** | **81.7 ± 4.10 c** | **12.2 ± 2.47 a** | 13.5 ± 0.75 a | 24.8 ± 2.27 a | 71.0 ± 10.38 a | **621.2 ± 141.6 a** | 2.88 ± 0.44 ab |
| **Low-input. 2023** | **Mv9kr1** | 84.4 ±9.6 b | 6.5 ± 2.1 b | 11.2 ± 1.4 a | 22.0 ± 1.9 a | 55.6 ± 13.9 a | 278.6 ± 78.5 b | 2.5 ± 0.5 a |
|  | **Mv Karizma** | 79.1 ± 4.0 b | 7.9 ± 2.6 ab | 11.8 ± 1.6 a | 20.7 ± 1.3 a | 47.6 ± 6.7 a | 276.7 ± 63.4 b | 2.3 ± 0.4 a |
|  | **CS** | 113.8 ± 5.7 a | 7.1 ± 1.9 b | 7.50 ± 0.6 b | 22.4 ± 2.6 a | 46.1 ± 8.5 a | 255.0 ± 90.5 b | 2.1 ± 0.4 a |
|  | **WT153397** | 77.9 ± 4.78 b | 10.6 ± 3.4 a | 12.4 ± 2.0 a | 22.2 ± 2.5 a | 53.1 ± 10.3 a | **409.4 ± 90.6 a** | 2.4 ± 0.4 a |
| **High-input. 2023** | **Mv9kr1** | 101.2 ± 6.0 b | 8.57 ± 2.51 c | 11.3 ± 1.0 a | 21.8 ± 1.9 a | 45.4 ± 5.8 a | 387.6 ± 81.24 b | 2.07 ± 0.3 a |
|  | **Mv Karizma** | 99.5 ± 6.4 b | 12.0 ± 1.5 b | 11.5 ± 0.9 a | 20.4 ± 0.5 a | 43.2 ± 6.6 a | 387.4 ± 68.6 b | 2.2 ± 0.4 a |
|  | **CS** | 117.9 ± 9.1 a | 7.8 ± 0.8 c | 8.4 ± 0.5 b | 22.0 ± 1.6 a | 47.3 ± 14.1 a | 305.6 ± 79.55 c | 2.2 ± 0.6 a |
|  | **WT153397** | **88.7 ± 4.4 c** | **16.3 ± 2.2 a** | 11.2 ± 0.5 a | 21.0 ± 0.7 a | 54.6 ± 4.0 a | **489.5 ± 83.8 a** | 2.6 ± 0.2 a |

**Supplementary Table S6** Average grain width (in mm), average grain length (in mm), and thousand grain weight (in grams) of two wheat parental genotypes (Mv9kr1 and Mv Karizma) and the WT153397 translocation line in the four growing seasons. Values are the means ± standard deviations of 3 measurements. Values with the same letters in a column per year do not differ significantly by Tukey’s post hoc test (α = 0.05).

| field trial | genotype | average grain width (mm) | average grain length (mm) | TGW (g) | yield/plant (g) |
| --- | --- | --- | --- | --- | --- |
| 2019 Low-input | Mv9kr1 | 3.51 ± 0.04 a | 6.39 ± 0.11 b | 38.57 ± 1.70 a | 12.38 ± 3.7 b |
|  | Mv Karizma | 3.3 ± 0.01 b | 6.63 ± 0.16 a | 39.27 ± 0.25 a | 17.24 ± 4.2 ab |
|  | WT153397 | 3.09 ± 0.06 c | 6.09 ± 0.24 b | 34.21 ± 2.0 b | 20.5 ± 5.2 a |
| 2021 Low-input | Mv9kr1 | 3.54 ± 0.1 a | 6.78 ± 0.34 a | 42.78 ± 3.19 a | 13.0 ± 3.9 b |
|  | Mv Karizma | 3.24 ± 0.03 b | 6.62 ± 0.09 a | 36.53 ± 2.58 b | 16.26 ± 5.26 ab |
|  | WT153397 | 2.92 ± 0.08 c | 6.02 ± 0.27 c | 32.1 ± 1.82 b | 19.79 ± 4.2 a |
| 2022 Low-input | Mv9kr1 | 3.59 ± 0.07 a | 6.69 ± 0.45 a | 43.95 ± 2.43 a | 16.11 ± 4.3 a |
|  | Mv Karizma | 3.28 ± 0.01 b | 6.6 ± 0.1 a | 39.72 ± 1.25 b | 11.57 ± 3.2 b |
|  | WT153397 | 3.0 ± 0.01 c | 5.84 ± 0.01 b | 29.08 ± 0.60 c | 15.23 ± 4.2 a |
| 2022 High -input | Mv9kr1 | 3.26 ± 0.01 a | 6.52 ± 0.05 a | 32.37 ± 0.72 a | 12.15 ± 2.7 b |
|  | Mv Karizma | 3.1 ± 0.03 b | 6.65 ± 0.03 a | 31.85 ± 0.29 a | 13.47 ± 1.43 b |
|  | WT153397 | 3.01 ± 0.03 c | 6.05 ± 0.11 b | 31.01 ± 0.89 a | 19.26 ± 5.4 a |
| 2023 Low- input | Mv9kr1 | 3.50 ± 0.03 a | 6.42 ± 0.03 a | 39.21 ± 1.55 a | 10.9 ± 5.7 b |
|  | Mv Karizma | 3.13 ± 0.02 b | 6.44 ± 0.04 a | 33.41 ± 1.41 b | 9.2 ± 2.1 b |
|  | WT153397 | 3.12 ± 0.03 b | 6.19 ± 0.04 b | 32.49 ± 1.58 b | 13.3 ± 2.9 a |
| 2023 High -input | Mv9kr1 | 3.35 ± 0.02 a | 6.68 ± 0.1 a | 35.4 ± 0.94 a | 13.72 ± 3.6 a |
|  | Mv Karizma | 3.28 ± 0.03 b | 6.63 ± 0.05 a | 37.62 ± 1.01 a | 14.57 ± 3.9 a |
|  | WT153397 | 2.95 ± 0.02 c | 5.99 ± 0.04 b | 29.95 ± 0.24 b | 14.66 ± 1.2 a |

**Supplementary Table S7** Morphological traits of the Mv9kr1wheat line, Mv Nádor elite wheat cultivar and WT153397 × Mv Nádor offsprings (F_5_ generation) in 2023 growing season in high-input nursery. Values with the same letters in a row do not differ significantly by Tukey’s post hoc test (α = 0.05). Values of WT153397 significantly different from the wheat genotypes have been highlighted by bold letters.

|  | Genotype | | |
| --- | --- | --- | --- |
|  | Mv9kr1 | MvNádor | WT153397 × MvNádor (F_5_) |
| Plant height (cm) | 101.2 ± 5.9 a | 80.1 ± 3.2 b | 76.4 ± 10.9 b |
| Number of spikes/plant | 8.57 ± 1.6 b | 7.64 ± 1.6 b | **16.43 ± 4.2 a** |
| Length of main spike (cm) | 11.3 ± 1.0 a | 10.9 ± 1.1 a | 11.3 ± 1.1 a |
| Number of spikelets/main spike | 21.8 ± 2.2 a | 18.9 ± 1.0 b | 20.1 ± 2.0 b |
| Number of Seeds/main spike | 45.4 ± 9.3 c | 62.4 ± 5.3 b | **76.1 ± 10.9 a** |
| Fertility (number of seeds/spikelet) | 2.07 ± 0.4 c | 3.3 ± 0.3 b | **3.9 ± 0.6 a** |
| Average grain width (mm) | 3.5 ± 0.03 a | 3.13 ± 0.03 b | 3.4 ± 0.14 a |
| Average grain length (mm) | 6.42 ± 0.03 c | 7.52 ± 0.02 b | **7.9 ± 0.36 a** |
| Thousand Grain Weight (g) | 39.21 ± 1.55 a | 31.4 ± 1.7 b | 43.71 ± 2.55 a |
